# Low Serum Cholesterol Level Is a Significant Prognostic Factor That Improves CLL-IPI in Chronic Lymphocytic Leukaemia

**DOI:** 10.3390/ijms24087396

**Published:** 2023-04-17

**Authors:** Rui Gao, Kaixin Du, Jinhua Liang, Yi Xia, Jiazhu Wu, Yue Li, Bihui Pan, Li Wang, Jianyong Li, Wei Xu

**Affiliations:** 1 Department of Hematology, The First Affiliated Hospital of Nanjing Medical University, Jiangsu Province Hospital, Collaborative Innovation Center for Cancer Personalized Medicine, Nanjing 210029, China; 2 Department of Endocrinology, The First Affiliated Hospital of Nanjing Medical University, Jiangsu Province Hospital, Nanjing 210029, China; 3 Oxford Centre for Diabetes, Endocrinology and Metabolism, University of Oxford, Churchill Hospital, Oxford OX3 7LE, UK

**Keywords:** chronic lymphocytic leukaemia, high-density lipoprotein cholesterol, low-density lipoprotein cholesterol, CLL-IPI, prognosis

## Abstract

Hypocholesterolaemia is associated with elevated cancer risk and mortality, yet the relation between chronic lymphocytic leukaemia (CLL) and serum lipid profile remains unclear. Our study aims to evaluate the prognostic value of cholesterol levels in CLL and develop a prognostic nomogram that incorporates lipid metabolism. We enrolled 761 newly diagnosed CLL patients and separated them into either derivation (n = 507) or validation (n = 254) cohorts. The prognostic nomogram was constructed through multivariate Cox regression analyses, with performance evaluated using C-index, the area under the curve, calibration, and decision curve analyses. Decreased total cholesterol (TC), high-density lipoprotein cholesterol (HDL-C), and low-density lipoprotein cholesterol (LDL-C) at diagnosis were significantly associated with worse time to first treatment (TTFT) and cancer-specific survival (CSS), and simultaneously, low HDL-C with low LDL-C was identified as an independent prognostic indicator for both TTFT and CSS. CLL patients achieving complete or partial remission post-chemotherapy had significantly increased TC, HDL-C, and LDL-C levels compared with the baseline, and post-therapeutic HDL-C and LDL-C elevation correlated with favourable survival. The prognostic nomogram augmenting the CLL international prognostic index with low cholesterol levels yielded higher predictive accuracy and discrimination capacity for both 3-year and 5-year CSS. In conclusion, cholesterol profiles can be used as a cheap and readily accessible tool for predicting prognosis in CLL practice.

## 1. Introduction

Chronic lymphocytic leukaemia (CLL) is the most common form of leukaemia in Western countries [1] characterized by relentless accumulation of mature B lymphocytes in the peripheral blood with a typical immunophenotype of CD5/CD19/CD20/CD23+ [2]. Although the majority of CLL patients are diagnosed at an asymptomatic early stage [3], due to the biological, genetic, and molecular heterogeneity of CLL patients, identification and refinement of prognostic indices are essential for risk-adapted management. During the last decade, a remarkable effort has been invested in developing new prognostic models, yet most studies do not fully represent the general CLL population and, therefore, have limited translational value [4]. The CLL international prognostic index (CLL-IPI) has been widely used in the era of chemoimmunotherapy. It was derived from a large meta-analysis with patients at all clinical stages [5], integrating five variables including age, clinical stage, *TP53* status, immunoglobulin heavy-chain variable genes (*IGHV*) mutational status, and β_2_-microglobulin (β_2_-MG). Another scoring system, the international prognostic score of early-stage CLL (IPS-E), was recently proposed to predict the likelihood of treatment requirements in early-stage CLL patients [6]. Introduction of B-cell receptor (BCR) signalling pathway inhibitors (ibrutinib, acalabrutinib, idelalisib, and duvelisib) and anti-apoptotic protein B-cell lymphoma 2 (BCL-2) inhibitors (venetoclax) has also reshaped the therapeutic landscape [7] and prognostic evaluation of CLL [8]. However, in developing countries, where genetic and molecular markers are generally expensive and technically challenging, it is critical to develop a cheap and readily accessible prognostic indicator to enable risk stratification in patients with CLL.

Cholesterol is indispensable for the proliferation of cancer cells, providing several essential biological functions: (1) maintenance of cell membrane structure [9]; (2) providing a platform for growth signalling proteins, such as the vascular endothelial growth factor receptor-1 (VEGFR-1) [10]; and (3) modulation of the cell cycle by intermediate cholesterol metabolites [11]. Hypocholesterolaemia, possibly due to increased demand and uptake of cholesterol esters, has been reported in patients with lung [12,13], gastrointestinal [14], thyroid [15], breast [16], ovarian [17], and prostate [18] cancers. In addition to solid tumours, decreased cholesterol levels have also been observed in haematological cancers [19], such as CLL [20], acute lymphocytic leukaemia [21], lymphomas [22], and multiple myeloma [23]. However, the prognostic significance of hypocholesterolaemia in these malignancies is less investigated. It is also surprising to find that there are few current CLL prognostic indicators or models utilising reprogramed lipid metabolism, particularly considering that CLL cells demonstrate increased uptake of cholesterol and primarily utilise oxidative phosphorylation of free fatty acids to satisfy the high metabolic demand required to proliferate [24]. Altered cholesterol levels are easily obtainable parameters that reflect the overall nutritional status of a patient and should, therefore, have significant potential to predict CLL survival.

The aims of our study were to (1) investigate the correlation between pre-diagnostic serum lipid profile (total cholesterol (TC), high-density lipoprotein cholesterol (HDL-C), low-density lipoprotein cholesterol (LDL-C), triglycerides (TG), and lipoprotein (a) (Lp(a))) and CLL clinical characteristics, (2) evaluate the prognostic value of cholesterol levels in CLL survival and treatment response, and (3) construct a prognostic nomogram, incorporating lipid metabolism, to validate the incremental predictive capability of cholesterol levels on CLL-IPI, thereby facilitating risk stratification in CLL patients.

## 2. Results

### 2.1. Correlation between Clinical Characteristics and Lipid Profile

A total of 761 newly diagnosed CLL patients were enrolled in our study and randomly divided into either derivation (n = 507) or internal validation cohorts (n = 254). Baseline demographic and clinical characteristics are summarized in Table 1, and no significant difference between the two cohorts was identified. The median follow-up was 76.3 months and 79.1 months for the derivation and internal validation cohorts, respectively. Among 761 patients, 543 received treatments and the regimens included fludarabine + cyclophosphamide ± rituximab (N = 188, 34.6%), bendamustine ± rituximab (N = 40, 7.4%), chlorambucil ± rituximab (N = 121, 22.3%), ibrutinib ± rituximab (N = 125, 23.0%), ibrutinib + fludarabine + cyclophosphamide + rituximab (N = 23, 4.2%), and other treatments (N = 46, 8.5%).

Regarding serum cholesterol levels across the entire cohort, 106 (13.9%) exhibited low TC (<3.00 mmol/L), 430 (56.5%) exhibited low HDL-C (<1.03 mmol/L), and 370 (48.6%) exhibited low LDL-C (<2.60 mmol/L). A synchronous decrease in HDL-C together with LDL-C was detected in 275 (36.1%) patients. As shown in Table 1, a consistent pattern of decreased TC, HDL-C, and LDL-C levels was identified to be correlated with the following clinical parameters: male sex, Binet stage B/C, high CLL-IPI, low Hb, platelet and albumin levels, and high β_2_-MG level. These were apparent in both derivation and internal validation cohorts. Additionally, none of the CLL treatment regimens were significantly associated with specific changes in lipid profile after post hoc correction for multiple comparisons (Table 1).

### 2.2. Serum Lipid Profile as a Significant Prognostic Factor in CLL

**Figure 1:**
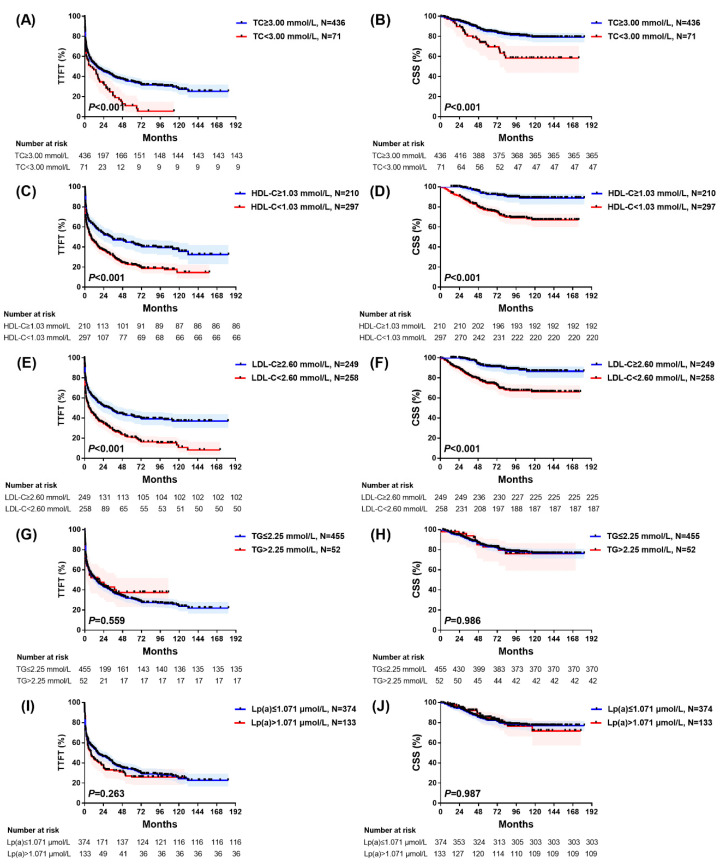
Kaplan–Meier survival curves stratified by serum lipid profile in the primary cohort. Time to first treatment (TTFT) and cancer-specific survival (CSS) in relation to total cholesterol (TC) (**A**,**B**), high-density lipoprotein cholesterol (HDL-C) (**C**,**D**), low-density lipoprotein cholesterol (LDL-C) (**E**,**F**), triglycerides (TG) (**G**,**H**), and lipoprotein (a) (Lp(a)) (**I**,**J**).

Within the derivation cohort, 355 (70.02%) patients underwent treatments, while the remaining 152 (29.98%) were managed according to a watch-and-wait approach. For mortality, 95 (18.74%) patients deceased before the end of the follow-up period, while the other 412 (81.26%) survived. As demonstrated in Figure 1, a significantly inferior time to first treatment (TTFT) was individually identified in patients with low TC (*p* < 0.001), LDL-C (*p* < 0.001), or HDL-C (*p* < 0.001). Similarly, decreased levels of TC (*p* < 0.001), LDL-C (*p* < 0.001), or HDL-C (*p* < 0.001) were all evidently associated with shorter cancer-specific survival (CSS). However, no significant difference was observed in survival outcomes stratified by TG (*p* = 0.559 for TTFT and *p* = 0.986 for CSS) and Lp(a) (*p* = 0.263 for TTFT and *p* = 0.987 for CSS). 

Using the aforementioned cut-off values for HDL-C and LDL-C, we categorized the derivation cohort into four subgroups: patients with synchronously low HDL-C and LDL-C (Group 1), only low HDL-C (Group 2), only low LDL-C (Group 3), and normal HDL-C and LDL-C (Group 4). Pairwise over strata analyses were performed, and comparing the survival of each pair of groups showed that patients with synchronously low HDL-C and LDL-C (Group 1) stood out by having significantly worse TTFT and CSS (Supplemental Figure S1 and Table S1). No significant differences were found in pairwise comparisons amongst the other three groups. These findings could suggest that dividing the derivation cohort according to whether patients had synchronously low HDL-C and LDL-C would be appropriate for further univariate and multivariate Cox regression models.

Clinical and laboratory variables of gender, age, Binet stage, Eastern cooperative oncology group (ECOG) performance status (PS), B symptoms, lipid profile, levels of lymphocyte count (ALC), haemoglobin (Hb), platelet count (PLT), lactate dehydrogenase (LDH), and β_2_-MG, together with cytogenetic and molecular parameters of *TP53* disruption, *ATM* deletion, *IGHV* unmutated status, and CD38 were initially included in the univariate Cox regression analyses of the derivation cohort. Factors with *p* < 0.05 further entered the multivariate Cox proportional hazards model using backward elimination (variance inflation factor (VIF) < 5 and tolerance > 0.2 for both TTFT and CSS) (Table 2). For TTFT, seven parameters remained statistically significant in the multivariate model: advanced Binet stage, B symptoms, decreased PLT, elevated LDH, *TP53* disruption, *IGHV* unmutated status, and simultaneously low HDL-C and LDL-C. For CSS, alongside low HDL-C and LDL-C, 5 other variables were selected including age > 65 years, advanced Binet stage, elevated β_2_-MG, *TP53* disruption, and *IGHV* unmutated status. These five variables are in accordance with parameters in CLL-IPI, which validates the prognostic efficacy and reproductivity of CLL-IPI. Thus, the presence of simultaneously low HDL-C and LDL-C is an independent prognostic indicator for both TTFT (hazard ratio (HR) = 1.488; 95% confidence interval (CI): 1.187–1.865; *p* = 0.001) and CSS (HR = 2.907; 95% CI: 1.848–4.572; *p* < 0.001) in CLL patients.

### 2.3. Post-Chemoimmunotherapeutic Cholesterol Fluctuation in Relation to Treatment Response and Prognosis

**Figure 2:**
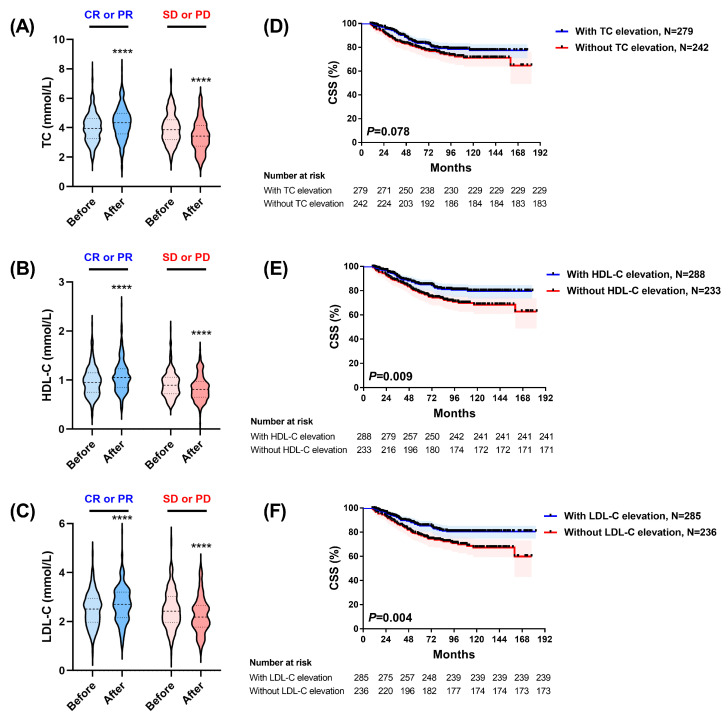
Post-chemoimmunotherapeutic cholesterol fluctuation in relation to treatment response and prognosis. (**A**–**C**) Pre- and post-therapeutic levels of total cholesterol (TC) (**A**), high-density lipoprotein cholesterol (HDL-C), (**B**) and low-density lipoprotein cholesterol (LDL-C) (**C**) in patients with complete remission (CR) or partial remission (PR) and stable disease (SD) or progressive disease (PD). (**D**–**F**) Comparison of survival between patients with and without TC (**D**), HDL-C (**E**), or LDL-C (**F**) elevation after completion of therapies. Abbreviations: TTFT: time to first treatment; CSS: cancer-specific survival. **** *p*-value < 0.0001.

To investigate the correlation between post-therapeutic changes in lipid profile and CLL response to treatment, we included 521 patients in the entire cohort who had completed full cycles of chemoimmunotherapy, while 22 further treated patients deceased before the response assessment and were, therefore, eliminated. Complete remission (CR) or partial remission (PR) was achieved in 301 (57.77%) patients, while stable disease (SD) or progressive disease (PD) was identified in 220 (42.23%) patients. As presented in Figure 2A–C, patients with CR or PR exhibited significantly increased post-therapeutic TC (4.02 ± 1.02 mmol/L to 4.31 ± 1.09 mmol/L, *p* < 0.001), HDL-C (0.97 ± 0.30 mmol/L to 1.08 ± 0.32 mmol/L, *p* < 0.001), and LDL-C (2.49 ± 0.70 mmol/L to 2.71 ± 0.77 mmol/L, *p* < 0.001) compared with the cholesterol levels at CLL diagnosis. By contrast, a significant reduction in TC (3.95 ± 1.03 mmol/L to 3.45 ± 1.07 mmol/L, *p* < 0.001), HDL-C (0.91 ± 0.26 mmol/L to 0.84 ± 0.26 mmol/L, *p* < 0.001), and LDL-C (2.50 ± 0.77 mmol/L to 2.26 ± 0.73 mmol/L, *p* < 0.001) was also observed in patients assessed as SD or PD. 

We then wondered whether the correlation between post-therapeutic cholesterol changes and response to treatment was specific to certain regimens. As shown in Supplemental Table S2, patients treated with regimens of fludarabine + cyclophosphamide ± rituximab or ibrutinib ± rituximab exhibited a consistent increase in TC, HDL-C, and LDL-C levels when evaluated as CR or PR. Conversely, in patients treated with chlorambucil ± rituximab, a marked decrease in lipid profile was significantly associated with SD or PD. To further determine whether biological and genetic aberrations may contribute to treatment response-related cholesterol fluctuation, we conducted subgroup analyses in patients with or without *TP53* disruption, *ATM* deletion, *IGHV* unmutated status, and CD38 ≥ 30% (Supplemental Table S3). Interestingly, an increase in cholesterol levels with CR or PR and a reduction with SD or PD were consistently observed across all subgroups, suggesting that post-therapeutic lipid changes were not dependent on these biological variables.

Subsequently, we analysed the prognostic value of elevation in post-therapeutic cholesterol levels using the Kaplan–Meier method. Interestingly, patients with increased HDL-C (*p* = 0.009) or LDL-C (*p* = 0.004) levels after completion of chemoimmunotherapy cycles presented with significantly favourable CSS in comparison with those that did not exhibit increased HDL-C or LDL-C (Figure 2E,F). Similarly, an effect approaching borderline significance was also observed regarding TC elevation (Figure 2D).

### 2.4. Construction and Prognostic Performance of Model_Lipo-IPI_

**Figure 3:**
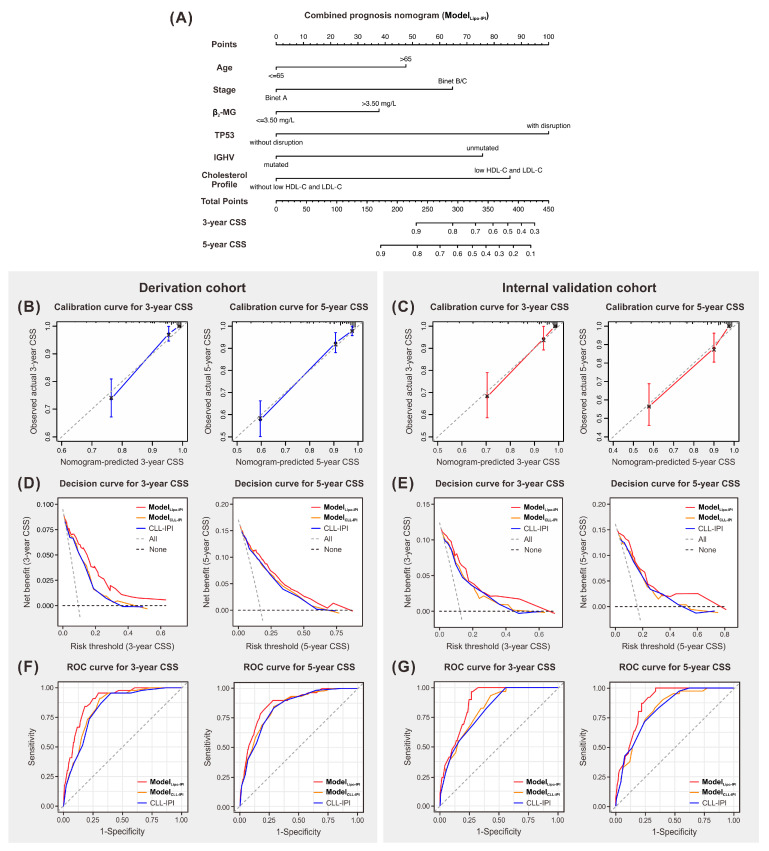
Nomogram and prognostic performance of Model_Lipo-IPI_ for cancer-specific survival (CSS). (**A**) A combined prognostic nomogram Model_Lipo-IPI_ for predicting CSS in CLL patients, including age, stage, β_2_-microglobulin (β_2_-MG) level, *TP53*, immunoglobulin heavy chain variable region (*IGHV*) status, and cholesterol profile as factors. (**B**,**C**) Calibration curves of Model_Lipo-IPI_ for predicting 3-year and 5-year CSS in the derivation (**B**) and internal validation (**C**) cohorts. (**D**,**E**) Decision curve analyses of different models for predicting 3-year and 5-year CSS in the derivation (**D**) and internal validation (**E**) cohorts. (**F**,**G**) Receiver operating characteristic (ROC) curves of different models for predicting 3-year and 5-year CSS in the derivation (**F**) and internal validation (**G**) cohorts. Abbreviations: HDL-C, high-density lipoprotein cholesterol; LDL-C, low-density lipoprotein cholesterol; CLL-IPI, international prognostic index for chronic lymphocytic leukaemia.

We developed a nomogram (Model_Lipo-IPI_) for CSS that integrates all the significant independent factors in the multivariate Cox regression analyses of the derivation cohort, including age, stage, β_2_-MG level, *TP53*, *IGHV* status, and cholesterol profile (Figure 3A). Each parameter was assigned a point based on the HR, and by adding up the total points, referencing the point scale, the probability of 3-year and 5-year CSS can be calculated. As displayed by calibration plots in Figure 3B,C, an optimal agreement was achieved between the prediction of the nomogram and observed actual CSS in both derivation and internal validation cohorts. 

To investigate the incremental prognostic value of the cholesterol profile, a reduced model was also constructed (Model_CLL-IPI_), which consisted of five parameters in CLL-IPI: age, stage, β_2_-MG level, *TP53*, and *IGHV* status. As shown in decision curve analyses of CSS in the derivation cohort (Figure 3D), Model_Lipo-IPI_ presented with a more desirable clinical net benefit compared with Model_CLL-IPI_ and, the gold standard, CLL-IPI. Furthermore, of all the models, Model_Lipo-IPI_ provided the most robust prognostic accuracy and capacity for discrimination capacity of CSS with a clear higher concordance index (C-index) and larger area under the curves (AUCs) of the time-dependent receiver operating characteristic (ROC) curves (3-year and 5-year) in the derivation cohort (Figure 3F and Table 3). The above findings were also verified by the internal validation cohort with consistent results (Figure 3E,G and Table 3). The success of Model_Lipo-IPI_ in obtaining the best prognostic performance suggests that the lipid profile adds power to CLL-IPI in predicting CSS outcomes. We also assessed the value of Model_Lipo-IPI_ in predicting TTFT using both derivation and internal validation cohorts. Model_Lipo-IPI_ was consistent with observed actual TTFT at 1-year and 3-year (Supplemental Figure S2A,B), whereas C-index and time-dependent ROC analyses did not demonstrate any significant improvement in prediction with Model_Lipo-IPI_ over the reduced Model_CLL-IPI_ (Supplemental Figure S2E,F and Table 3).

Risk stratification analyses of the entire cohort demonstrated that Model_Lipo-IPI_ was able to successfully classify patients into low, low-intermediate, high-intermediate, and high-risk groups according to respective quantiles (Supplemental Figure S3). Significant differences between each pair of risk categories were identified by pairwise comparison for both TTFT and CSS, suggesting Model_Lipo-IPI_ could accurately differentiate the survival outcomes of CLL patients (Supplemental Table S4).

### 2.5. Prognostic Value of Cholesterol Levels and Model_Lipo-IPI_ in the Era of Targeted Therapies

Of 543 treated patients in the entire cohort, 158 (29.1%) received novel targeted therapies, including ibrutinib ± rituximab, ibrutinib + fludarabine + cyclophosphamide + rituximab, and part of other treatments, while the remaining 385 (70.9%) were treated with traditional chemoimmunotherapies, such as fludarabine + cyclophosphamide ± rituximab, bendamustine ± rituximab, chlorambucil ± rituximab, and part of other treatments. To validate the stability of low cholesterol level as a prognostic predictor when regimen type is considered as a potential confounder, we conducted univariate and multivariate analyses for CSS, including an additional factor of whether patients received targeted therapies in 543 treated patients. After adjusting for treatment type, low HDL-C with low LDL-C still remained a significant factor associated with unfavourable CSS (HR = 3.437; 95% CI: 2.302–5.131; *p* < 0.001; Supplemental Table S5).

To further verify the efficacy of utilising Model_Lipo-IPI_ in the era of targeted therapies, we applied the developed nomogram in 158 patients treated with novel targeted regimens. As presented in Supplemental Figure S4 and Table S6, prognostic nomogram Model_Lipo-IPI_ augmenting CLL-IPI with low cholesterol levels still yielded better predictive accuracy and discrimination capacity for 3-year and 5-year CSS with significantly higher C-index, larger AUCs, and most desirable clinical net benefit compared with Model_CLL-IPI_ or CLL-IPI alone. Taken together, the application of low cholesterol levels and Model_Lipo-IPI_ may still improve prognostic prediction and risk stratification in the era of targeted therapies.

### 2.6. T Cell Subset Counts in Relation to Cholesterol Levels

**Figure 4:**
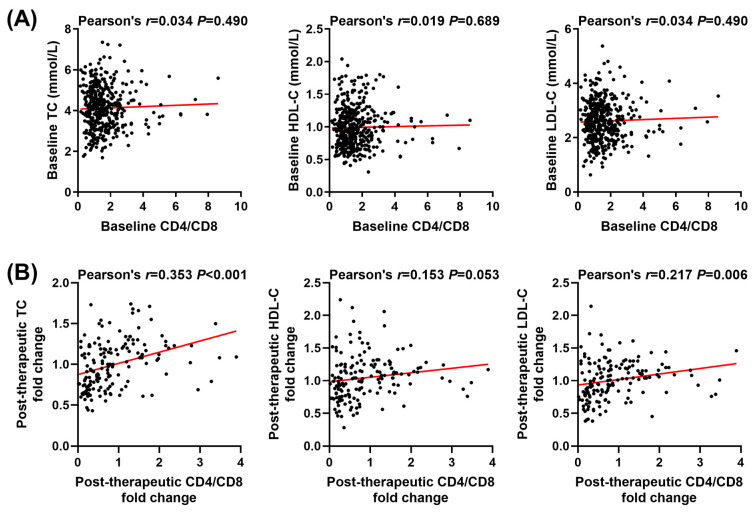
T cell subset counts in relation to cholesterol levels. (**A**) Correlation between baseline CD4/CD8 ratio and total cholesterol (TC), high-density lipoprotein cholesterol (HDL-C), and low-density lipoprotein cholesterol (LDL-C) levels; (**B**) Correlation between post-therapeutic fold change of CD4/CD8 ratio and fold change of TC, HDL-C, and LDL-C.

The development of CLL is characterized by a plethora of T cell abnormalities, including T cell expansion, differentiation, and activation [25]. Given that changes in cholesterol metabolism can significantly impact T cell function [26], we hypothesized that the link between low cholesterol levels and poor prognosis in CLL may be attributable to T cell subset variations. Baseline values of lymphocyte subset count were available for 427 patients in the entire dataset, of which 161 treated patients had complete pre- and post-therapeutic results. Although no specific pattern was observed between baseline CD4/CD8 ratio and cholesterol levels (TC, HDL-C, and LDL-C) (Figure 4A), a significant positive correlation was identified between post-therapeutic fold change of CD4/CD8 ratio and fold change of TC (*p* < 0.001) and LDL-C (*p* = 0.006) (Figure 4B). These data suggest that patients who experience a marked elevation in cholesterol levels are more likely to have a dramatic increase in CD4/CD8 ratio.

## 3. Discussion

To our knowledge, this is the first study to assess the prognostic value of the lipid profile in CLL patients and to demonstrate that hypocholesterolaemia is an independent risk factor associated with inferior survival. Although the relationship between low cholesterol and haematological cancer, as either cause or effect, has not been established, an altered systemic lipid profile could still serve as a useful biochemical or prognostic marker for patients with newly diagnosed CLL.

Hypocholesterolaemia may present during the course of oncohaematological disorders. Decreased levels of TC, HDL-C and LDL-C were reported in patients with newly diagnosed CLL [20], multiple myeloma [23], and lymphoma [27] and were found to be dependent on disease stage or progress. The drop in HDL-C level was even evident several years prior to lymphoma diagnosis, indicating an early role of cholesterol-related pathways in lymphomagenesis [27]. Low HDL-C level was also significantly associated with an increased risk of haematological malignancy [19]; specifically, each 5 mg/dL reduction in HDL-C corresponded with a 15% elevation in non-Hodgkin lymphoma risk [22]. Similarly, for LDL-C, a Mendelian randomization study identified low plasma LDL-C (below the 10th percentile) was strongly related to a 95% increase in haematological cancer risk [28].

Epidemiological association between the cholesterol paradigm and prognosis of haematological cancer was primarily established for HDL-C over the last decade. Correlation between decreased levels of HDL-C and poor clinical outcomes has been consistently reported in follicular lymphoma [29], extranodal natural killer/T cell lymphoma [30], malignant lymphoma, and adult T-cell leukaemia-lymphoma [31], whilst the prognostic potential of the combination of HDL-C together with LDL-C has rarely been investigated. Our findings suggest low TC, HDL-C, and LDL-C levels at diagnosis were clearly associated with unfavourable TTFT and CSS in CLL patients. As for other haematological malignancies, our group showed in previous studies that concurrently low HDL-C and LDL-C could be used as an independent prognostic factor for survival of diffuse large B cell lymphoma [32] and peripheral T-cell lymphoma [33].

Currently, the mechanisms underlying the association between hypocholesterolaemia and CLL development are not well understood. Dysregulation of cholesterol homeostasis has been suggested to participate in certain forms of carcinogenesis, especially in leukaemia cells, which display increased synthesis and uptake of cholesterol to satisfy their high turnover rate [34]. HDL-C is known to have a protective role against cancer through anti-inflammatory and anti-oxidative properties. Inflammatory pathways activated by immune factors are part of the mechanisms that lead to leukemogenesis [35]. The anti-inflammatory action of HDL-C may be mediated via the inhibition of cytokine-induced expression of endothelial cell adhesion molecules and suppression of the chemotactic response of monocytes and lymphocytes [36,37]. HDL-C can also help revert immune escape by reducing myeloid-derived suppressor cells, thus improving the recruitment of M1 tumour-associated macrophages and cytotoxic CD8^+^ T cells in the tumour microenvironment [38,39]. Additionally, HDL-C may also protect the integrity of lymphocytes by counteracting intracellular oxidative stress [40,41]. 

Alternative to a causative role, low plasma HDL-C level could also be an epiphenomenon of cancer presence. Tumour cells show enhanced expression of the scavenger receptor class B type 1, an HDL-C receptor, that facilitates cholesteryl esters uptake from HDL-C into the cytoplasm [42] and reduced expression of the ATP binding cassette transporter A1, which is involved in exporting cholesterol from peripheral and cancer cells [43]. These together contribute to the reduction in plasma HDL-C levels.

As for LDL-C, Benn et al. reported that although low plasma LDL-C correlated with an increased risk of cancer, this was not the case for a patient with genetically decreased LDL-C [28]. This indicates that low LDL-C concentration per se does not cause cancer but is more likely due to concomitant nutritional insufficiencies that occur as cancer progresses. The LDL receptor was found to be overexpressed in various malignancies, promoting LDL-C uptake and new membrane synthesis in order to meet the demand of cancer cells [44]. Furthermore, increased reactive oxygen species levels occurring during an inflammatory state can lead to the oxidation of LDL to oxidized LDL (ox-LDL). This results in a subsequent decrease in circulating LDL as ox-LDL is taken up by macrophages at the site of inflammation [45].

Richter’s transformation is a paradigmatic evolution of CLL into a highly aggressive large B cell lymphoma conferring a dismal prognosis [46]. Our study failed to detect an association between Richter’s syndrome and low cholesterol levels (Table 1) possibly due to the following reasons: (1) the limited number of Richter’s transformation cases identified in the entire dataset (47 out of 761 patients) that could affect the statistical power; and (2) the fact Richter’s transformed cells exhibited a more glycolytic phenotype with increased ^18^F-fluorodeoxyglucose uptake as compared with CLL cells [47,48]. This implies that these cells may rely more on glucose metabolism to sustain rapid proliferation rather than increased cholesterol uptake as a source of nutrients. 

Attempts have been made to elucidate the mechanisms underlying the post-chemotherapeutic cholesterol changes during the course of haematological malignancies. Alexopoulous et al. described this phenomenon as a reversal of an aberrant lipid profile secondary to malignancy after effective chemotherapy treatment [49]. Kuliszkiewicz-Janus et al. used ^31^P-magnetic resonance spectroscopy spectra to analyse the phospholipid changes in neoplastic cells and found that serum cholesterol measures returned to normal during remission in leukaemia and lymphoma [50,51]. Consistently, our study observed significant post-chemotherapeutic increases in TC, HDL-C, and LDL-C among CLL patients responding favourably to treatment, which correlated with better survival. We noticed that the association between changes in cholesterol levels and response to therapy was specific to certain treatment regimens but independent of the biological characteristics of CLL patients. Additionally, a positive correlation was further identified between post-therapeutic fold changes of CD4/CD8 ratio and fold changes of TC and LDL-C, indicating a potential role of T-cell-mediated-immune response in cholesterol level fluctuation. Together, these findings suggest that the patient’s lipid profile can serve as a biomarker of tumour activity, and longitudinal measurement of cholesterol may be beneficial for early detection of CLL relapse.

A remarkable effort has been made to develop new prognostic models using either weighed scoring or nomograms in the CLL patient population over the past five years. Although current models (i.e., CLL-IPI) have taken clinical, biological, and genetic irregularities into consideration, the importance of nutritional status or tumour metabolism during leukemogenesis has been overlooked. Based on the multivariate analyses for CSS, we constructed a new nomogram Model_Lipo-IPI_ including a lipid protein paradigm for CLL prognostic stratification. The reliability of this nomogram was verified by calibration plots, decision curves, C-index, and time-dependent AUCs in the derivation, internal validation, and ibrutinib validation cohorts. These suggest that the Model_Lipo-IPI_ can be a valuable tool for evaluating prognosis following initial diagnosis, promoting personalized treatment and guiding follow-up both in the era of chemoimmunotherapies and targeted therapies. Therefore, cholesterol profiles can be utilised as a cheap and accessible tool, delivering great benefits to CLL patients in clinical practice. 

Nevertheless, this study has several limitations. First, as with all epidemiological studies, we could not establish a causal relationship between the lipid profile and CLL development. Access to patients’ lipid trajectories prior to CLL diagnosis would likely offer insight into the role cholesterol plays in carcinogenesis. Second, this study was conducted in a Chinese population, thus further validation would be required to be utilised for other ethnic groups or communities. Third, our study was built on a single-centred retrospective cohort. Although the large size of the cohort and the internal validation enhances the reliability of our results, external validation would be necessary to further interpret its clinical application. Fourth, we acknowledged that the limited use of novel targeted therapy in our study population due to the late approval of ibrutinib by the China Food and Drug Administration may have implications for the generalisability of our findings. Therefore, further studies with a larger cohort are needed to validate the prognostic value of hypocholesterolaemia in the era of targeted therapies.

## 4. Materials and Methods

### 4.1. Ethics and Consent

This study was approved by the Ethics Committee of the First Affiliated Hospital of Nanjing Medical University (approval number: 2022-SR-312; approval date: 10 May 2022), and a waiver from informed consent was granted. All procedures performed in this study were in accordance with the 1964 Helsinki Declaration and its later amendments.

### 4.2. Patients

A total of 761 eligible patients with newly diagnosed CLL between January 2007 and January 2021 from the First Affiliated Hospital of Nanjing Medical University were retrospectively enrolled at initial diagnosis of CLL. The diagnoses were made based on the International Workshop on CLL-National Cancer Institute (IWCLL-NCI) criteria. Exclusion criteria included: (1) patients with incomplete clinical information, laboratory results, or follow-up data; (2) patients recruited after January 2021 to minimize the bias in TTFT and CSS caused by short follow-up time; (3) patients with prior malignancy; and (4) patients deceased due to accident or comorbidities unrelated to CLL to assess the prognostic value on CLL-specific mortality. 

### 4.3. Data Collection

Baseline demographic and clinical characteristics at diagnosis, including sex, age, Binet stage, ECOG PS, B symptoms, Richter transformation, and CLL-IPI, were retrieved from medical records. Laboratory data of ALC, Hb, PLT, LDH, albumin, β_2_-MG, and C-reactive protein (CRP) levels and lymphocyte subset counts within 24 h after the first admission were accessible from the hospital-based laboratory service.

Fluorescence in situ hybridization analysis was performed using fluorescent-labelled probes LSI ATM (11q22) and LSI p53 (17p13) (Vysis, Downers Grove, IL, USA) to detect del(11q22) and del(17p13), respectively [52]. Sanger sequencing of *TP53* (exons 4–9) was conducted as previously described [53]. We refer to the cohort with *TP53* mutation and/or del(17p13) as *TP53* disruption. Detection of *IGHV* mutation was performed as reported previously [54], and the 98% cut-off of germline homology is used to dichotomize *IGHV* mutational status. Immunophenotyping of CD38 was detected via flow cytometry, with the cut-off point for positivity set at 30% [55].

Serum lipid profile of TC (normal value range, 3.00–5.70 mmol/L), HDL-C (normal value range, 1.03–1.55 mmol/L), LDL-C (normal value range, 2.60–4.10 mmol/L), TG (normal value range, 0.00–2.25 mmol/L), and Lp(a) (normal value range, 0.00–1.017 μmol/L) was sampled within the same timeframe (6:00 am–8:00 am) after overnight fast. Because no clearly defined cut-off for lipid level was previously identified in relation to cancer prognosis and the manufacturer’s standards differ between medical facilities, we used the lower limits of normal TC (<3.00 mmol/L and ≥3.00 mmol/L), HDL-C (<1.03 mmol/L and ≥1.03 mmol/L), and LDL-C (<2.60 mmol/L and ≥2.60 mmol/L), as well as the upper limits of TG (≤2.25 mmol/L and >2.25 mmol/L) and Lp(a) (≤1.017 μmol/L and >1.017 μmol/L) as the cut-off for further analyses.

### 4.4. Follow-Up and Outcome Measures

The follow-up events included TTFT and CSS. TTFT is defined as the period from initial diagnosis to first-line treatment. CSS is calculated as the interval between diagnosis and CLL-specific death or the end of follow-up. Cause of death coded as 2A82.0 based on the International Classification of Diseases, 11th revision (ICD-11) was classified as CLL-specific death. The 761 patients enrolled were followed up for 1–181 months until December 2021, with a median follow-up time of 77.5 months. 

Assessment of response, including physical examination, evaluation of blood and bone marrow, and CT/PET-CT scan was performed approximately 2 months after completion of therapies. In addition, serum lipid levels and lymphocyte subset counts were measured at this time. 

### 4.5. Model Construction and Validation

Cases were randomly assigned (at a ratio of 2:1) into the derivation (n = 507) and internal validation cohorts (n = 254) by setting the seed in R (version 4.2.0). Using data from the derivation cohort, univariate and multivariate Cox regression models with HR and 95% CI were applied to assess the independent contribution of each factor and select variables for TTFT and CSS prediction. Specifically, each variable was first screened in the univariate model. Then, potential interactions between selected significant variables (*p* < 0.05) were examined by multiple collinearity diagnoses using VIF and tolerance value, before entering the multivariate model. Variables that demonstrate statistical significance in the multivariate Cox proportional hazard model (*p* < 0.05) were then chosen to construct the prognostic prediction model. 

Based on the multivariate Cox analysis, a combined prognosis nomogram Model_Lipo-IPI_, including lipid profile, was formulated to predict the 3- and 5-year CSS. Additionally, Model_CLL-IPI_ without lipid data was also developed using multivariate Cox regression in the derivation cohort to evaluate the incremental prognostic value of simultaneously low HDL-C and LDL-C. The performance of proposed models was assessed in both derivation and internal validation cohorts by Harrell’s C-index and AUC derived from the time-dependent ROC analysis. The calibration curves (1,000 bootstrap resamples) were plotted to evaluate the agreement between observed actual survival and the nomogram-predicted survival. Furthermore, decision curve analysis (DCA) was employed to assess net benefit of the nomogram in clinical context.

### 4.6. Statistical Analyses

Statistical analyses were performed using SPSS version 25.0 (SPSS, Chicago, IL, USA) and R version 4.2.0 with “Hmisc”, “rms”, “rmda”, “ggplot”, “ggDCA”, “survival”, “survcomp”, “compareC”, “survivalROC”, and “timeROC” packages. Differences in categorical variables (displayed as percentage) were compared using the Pearson χ^2^ test or Fisher’s exact test. Differences in continuous variables (displayed as mean ± standard deviation) between two groups were assessed by two-tailed Student’s *t*-test while for differences between more groups one-way ANOVA followed by a post hoc test was used. Underlying assumptions for the *t*-test and one-way ANOVA were previously assessed, including the normality test and the homogeneity test of variances. If the above assumptions were not met, Mann–Whitney *U* test was performed instead. Correlations were quantified using Pearson’s *r* or the Spearman test for parametric and nonparametric data analyses, respectively. Survival curves were drawn using Kaplan–Meier method and differences were computed by the log-rank test. A two-sided *p* < 0.05 is considered statistically significant.

## 5. Conclusions

In summary, we demonstrated that decreased levels of TC, HDL-C, and LDL-C at CLL diagnosis were significantly associated with worse TTFT and CSS. We also showed that simultaneously low HDL-C and low LDL-C was independent prognostic indicator for both TTFT and CSS. CLL patients who achieved CR or PR post-chemotherapy had significantly increased TC, HDL-C, and LDL-C levels compared with the levels at diagnosis, and elevation of either HDL-C or LDL-C correlated with favourable survival. The prognostic nomogram we developed, by augmenting CLL-IPI with low cholesterol levels, yielded higher predictive accuracy and discrimination capacity for 3-year and 5-year CSS in both derivation and internal validation cohorts. The application of this model has the ability, therefore, to significantly improve risk stratification and optimize the management of CLL patients.

## Figures and Tables

**Table 1:** Difference in serum cholesterol levels stratified by clinical characteristics in the enrolled CLL patients.

Variables		Derivation Cohort (N = 507)							Validation Cohort (N = 254)								
		Total	TC (mmol/L)	*p*-Value	HDL-C (mmol/L)	*p*-Value	LDL-C (mmol/L)	*p*-Value		Total	TC (mmol/L)	*p*-Value	HDL-C (mmol/L)	*p*-Value	LDL-C (mmol/L)	*p*-Value	
Clinical variables																	
Gender	Male	329	3.90 ± 1.01	<0.001	0.93 ± 0.27	<0.001	2.48 ± 0.76	<0.001		177	3.99 ± 1.00	0.003	0.97 ± 0.31	0.008	2.51 ± 0.73	0.019	
	Female	178	4.58 ± 1.16		1.08 ± 0.31		2.87 ± 0.82			77	4.41 ± 1.06		1.08 ± 0.31		2.74 ± 0.70		
Age	≤65 years	317	4.26 ± 1.11	0.002	1.00 ± 0.29	0.210	2.69 ± 0.79	0.005		175	4.11 ± 1.00	0.860	1.00 ± 0.30	0.559	2.57 ± 0.71	0.790	
	>65 years	190	3.95 ± 1.10		0.96 ± 0.29		2.49 ± 0.80			79	4.14 ± 1.12		1.02 ± 0.33		2.60 ± 0.76		
Binet stage	A	157	4.55 ± 1.02	<0.001	1.10 ± 0.29	<0.001	2.88 ± 0.73	<0.001		78	4.38 ± 0.97	0.007	1.11 ± 0.28	0.001	2.71 ± 0.66	0.049	
	B/C	350	3.96 ± 1.11		0.93 ± 0.28		2.50 ± 0.81			176	4.00 ± 1.04		0.96 ± 0.32		2.52 ± 0.75		
ECOG PS	0–1	441	4.17 ± 1.14	0.169	0.99 ± 0.30	0.250	2.64 ± 0.81	0.108		224	4.13 ± 1.02	0.638	1.01 ± 0.31	0.326	2.58 ± 0.72	0.880	
	>1	66	3.96 ± 0.92		0.95 ± 0.26		2.47 ± 0.72			30	4.04 ± 1.11		0.95 ± 0.32		2.60 ± 0.78		
Symptoms	No B symptoms	410	4.18 ± 1.12	0.149	1.00 ± 0.30	0.020	2.64 ± 0.80	0.128		195	4.15 ± 1.03	0.403	1.02 ± 0.32	0.068	2.60 ± 0.74	0.491	
	B symptoms	97	3.99 ± 1.08		0.92 ± 0.24		2.51 ± 0.79			59	4.02 ± 1.03		0.94 ± 0.29		2.52 ± 0.70		
Richter transformation	Absence	471	4.14 ± 1.11	0.764	0.99 ± 0.29	0.053	2.61 ± 0.80	0.758		243	4.12 ± 1.04	0.996	1.00 ± 0.31	0.559	2.58 ± 0.73	0.992	
	Presence	36	4.19 ± 1.20		0.89 ± 0.27		2.66 ± 0.90			11	4.12 ± 0.95		1.06 ± 0.31		2.58 ± 0.63		
CLL-IPI	0–3	285	4.37 ± 1.10	<0.001	1.06 ± 0.30	<0.001	2.76 ± 0.79	<0.001		141	4.32 ± 0.98	0.001	1.07 ± 0.30	<0.001	2.71 ± 0.69	0.002	
	4–10	222	3.85 ± 1.07		0.89 ± 0.25		2.44 ± 0.78			113	3.87 ± 1.05		0.92 ± 0.30		2.42 ± 0.74		
ALC	≤50 × 10^9^/L	393	4.20 ± 1.15	0.039	1.01 ± 0.30	<0.001	2.64 ± 0.83	0.201		202	4.18 ± 1.03	0.059	1.04 ± 0.31	<0.001	2.60 ± 0.72	0.378	
	>50 × 10^9^/L	114	3.95 ± 0.95		0.88 ± 0.24		2.53 ± 0.70			52	3.88 ± 1.04		0.87 ± 0.27		2.50 ± 0.74		
Hb	<100 g/L	106	3.54 ± 1.02	<0.001	0.85 ± 0.27	<0.001	2.27 ± 0.75	<0.001		53	3.40 ± 1.03	<0.001	0.81 ± 0.29	<0.001	2.15 ± 0.73	<0.001	
	≥100 g/L	401	4.30 ± 1.09		1.02 ± 0.29		2.71 ± 0.79			201	4.31 ± 0.95		1.06 ± 0.29		2.69 ± 0.69		
PLT	<100 × 10^9^/L	139	3.75 ± 1.10	<0.001	0.93 ± 0.28	0.006	2.37 ± 0.82	<0.001		65	3.62 ± 1.00	<0.001	0.90 ± 0.34	0.001	2.27 ± 0.77	<0.001	
	≥100 × 10^9^/L	368	4.29 ± 1.09		1.01 ± 0.29		2.71 ± 0.78			189	4.29 ± 0.99		1.04 ± 0.29		2.69 ± 0.68		
LDH	≤ULN (271 U/L)	394	4.19 ± 1.10	0.052	1.01 ± 0.29	0.002	2.64 ± 0.78	0.151		204	4.17 ± 1.06	0.092	1.03 ± 0.31	0.041	2.61 ± 0.75	0.216	
	>ULN (271 U/L)	113	3.96 ± 1.15		0.91 ± 0.30		2.52 ± 0.86			50	3.90 ± 0.90		0.92 ± 0.29		2.47 ± 0.64		
Albumin	<LLN (3.50 g/dL)	204	3.83 ± 1.08	<0.001	0.90 ± 0.26	<0.001	2.42 ± 0.80	<0.001		91	3.62 ± 1.02	<0.001	0.86 ± 0.28	<0.001	2.29 ± 0.68	<0.001	
	≥LLN (3.50 g/dL)	303	4.35 ± 1.09		1.04 ± 0.30		2.75 ± 0.78			163	4.40 ± 0.93		1.09 ± 0.30		2.74 ± 0.70		
β_2_-MG	≤3.50 mg/L	301	4.37 ± 1.09	<0.001	1.06 ± 0.30	<0.001	2.73 ± 0.78	<0.001		138	4.37 ± 0.95	<0.001	1.12 ± 0.32	<0.001	2.71 ± 0.69	<0.001	
	>3.50 mg/L	206	3.80 ± 1.07		0.87 ± 0.24		2.45 ± 0.80			116	3.82 ± 1.05		0.87 ± 0.25		2.42 ± 0.74		
CRP	≤ULN (1 mg/dL)	391	4.27 ± 1.08	<0.001	1.02 ± 0.29	<0.001	2.69 ± 0.79	<0.001		212	4.16 ± 0.99	0.182	1.04 ± 0.31	<0.001	2.59 ± 0.71	0.451	
	>ULN (1 mg/dl)	116	3.71 ± 1.13		0.86 ± 0.26		2.36 ± 0.80			42	3.93 ± 1.22		0.85 ± 0.26		2.50 ± 0.80		
Treatments	Fludarabine + cyclophosphamide ± rituximab	120	3.91 ± 1.03	0.907	0.93 ± 0.29	0.412	2.47 ± 0.70	0.937		68	4.02 ± 1.03	0.737	0.94 ± 0.27	0.195	2.55 ± 0.74	0.414	
	Bendamustine ± rituximab	26	4.06 ± 0.90		1.01 ± 0.28		2.55 ± 0.58			14	3.66 ± 0.84		0.85 ± 0.29		2.15 ± 0.53		
	Chlorambucil ± rituximab	79	4.02 ± 0.99		0.92 ± 0.26		2.46 ± 0.71			42	3.93 ± 0.97		0.94 ± 0.30		2.48 ± 0.67		
	Ibrutinib ± rituximab	85	3.95 ± 1.02		0.94 ± 0.26		2.49 ± 0.76			40	4.09 ± 0.84		1.00 ± 0.33		2.57 ± 0.61		
	Ibrutinib + fludarabine + cyclophosphamide + rituximab	16	4.20 ± 1.37		1.06 ± 0.35		2.66 ± 0.93			7	3.79 ± 0.78		0.96 ± 0.20		2.33 ± 0.54		
	Other treatments	29	3.98 ± 1.26		0.93 ± 0.25		2.51 ± 0.95			17	4.07 ± 1.06		1.11 ± 0.41		2.42 ± 0.78		
Biological variables																	
*TP53* disruption	Absence	384	4.22 ± 1.10	0.007	1.01 ± 0.30	<0.001	2.67 ± 0.79	0.008		209	4.19 ± 1.01	0.013	1.02 ± 0.30	0.138	2.63 ± 0.71	0.012	
	Presence	123	3.91 ± 1.15		0.90 ± 0.26		2.45 ± 0.82			45	3.78 ± 1.08		0.94 ± 0.37		2.33 ± 0.78		
*ATM* deletion	Absence	441	4.16 ± 1.15	0.370	0.99 ± 0.30	0.227	2.62 ± 0.82	0.640		207	4.14 ± 1.02	0.553	1.02 ± 0.31	0.141	2.59 ± 0.73	0.536	
	Presence	66	4.03 ± 0.86		0.94 ± 0.25		2.57 ± 0.65			47	4.04 ± 1.10		0.94 ± 0.30		2.52 ± 0.71		
*IGHV*	Unmutated	194	4.03 ± 1.02	0.091	0.93 ± 0.27	0.002	2.56 ± 0.75	0.217		110	3.98 ± 0.94	0.062	0.95 ± 0.30	0.020	2.50 ± 0.69	0.107	
	Mutated	313	4.21 ± 1.16		1.02 ± 0.30		2.65 ± 0.83			144	4.23 ± 1.09		1.04 ± 0.31		2.64 ± 0.75		
CD38	<30%	373	4.10 ± 1.07	0.203	0.99 ± 0.29	0.370	2.60 ± 0.78	0.483		185	4.11 ± 1.09	0.733	1.01 ± 0.32	0.704	2.55 ± 0.75	0.263	
	≥30%	134	4.25 ± 1.23		0.96 ± 0.29		2.66 ± 0.87			69	4.16 ± 0.89		0.99 ± 0.29		2.66 ± 0.66		

Abbreviations: CLL, chronic lymphocytic leukaemia; TC, total cholesterol; HDL-C, high-density lipoprotein cholesterol; LDL-C, low-density lipoprotein cholesterol; ECOG, eastern cooperative oncology group; PS, performance status; IPI, international prognostic index; ALC, absolute lymphocytic count; Hb, haemoglobin; PLT, platelet; LDH, lactate dehydrogenase; β_2_-MG, β_2_-microglobulin; CRP, C-reactive protein; *IGHV*, immunoglobulin heavy chain variable region; ULN, upper limit of normal; LLN, lower limit of normal.

**Table 2:** Univariate and multivariate analyses of TTFT and CSS in the derivation cohort.

Variables	TTFT				CSS			
	Univariate Analyses		Multivariate Analyses		Univariate Analyses		Multivariate Analyses	
	HR (95% CI)	*p*-Value	HR (95% CI)	*p*-Value	HR (95% CI)	*p*-Value	HR (95% CI)	*p*-Value
Male	1.228 (0.984–1.532)	0.070	–	–	1.501 (0.961–2.345)	0.074	–	–
Age > 65 years	0.956 (0.769–1.188)	0.684	–	–	1.879 (1.257–2.810)	0.002	1.806 (1.202–2.714)	0.004
Binet B/C	3.242 (2.483–4.234)	<0.001	2.170 (1.619–2.909)	<0.001	4.751 (2.391–9.441)	<0.001	2.236 (1.111–4.503)	0.024
ECOG PS > 1	0.991 (0.729–1.348)	0.956	–	–	1.589 (0.951–2.655)	0.077	–	–
B symptoms	2.304 (1.807–2.939)	<0.001	1.856 (1.450–2.375)	<0.001	1.045 (0.632–1.728)	0.863	–	–
ALC > 50 × 10^9^/L	1.637 (1.294–2.072)	<0.001	1.190 (0.935–1.516)	0.158	1.405 (0.904–2.183)	0.131	–	–
Hb < 100 g/L	2.108 (1.663–2.672)	<0.001	1.038 (0.793–1.358)	0.786	1.820 (1.176–2.818)	0.007	0.904 (0.573–1.427)	0.665
PLT < 100 × 10^9^/L	2.138 (1.712–2.670)	<0.001	1.377 (1.084–1.748)	0.009	1.630 (1.079–2.462)	0.020	0.935 (0.601–1.453)	0.764
LDH > ULN (271 U/L)	2.161 (1.711–2.730)	<0.001	1.464 (1.140–1.881)	0.003	2.144 (1.409–3.262)	<0.001	0.925 (0.585–1.463)	0.740
β_2_-MG > 3.50 mg/L	1.837 (1.489–2.266)	<0.001	1.000 (0.790–1.266)	1.000	3.015 (1.970–4.614)	<0.001	1.599 (1.032–2.479)	0.036
*TP53* disruption	2.353 (1.867–2.966)	<0.001	1.333 (1.032–1.721)	0.028	5.907 (3.913–8.918)	<0.001	3.468 (2.271–5.295)	<0.001
*ATM* deletion	1.149 (0.849–1.555)	0.369	–	–	1.129 (0.616–2.068)	0.696	–	–
*IGHV* unmutated	2.028 (1.642–2.505)	<0.001	1.462 (1.163–1.839)	0.001	3.523 (2.312–5.369)	<0.001	2.564 (1.665–3.948)	<0.001
CD38 ≥ 30%	1.363 (1.086–1.712)	0.008	1.067 (0.843–1.352)	0.588	1.261 (0.811–1.960)	0.303	–	–
Low HDL-C and LDL-C	2.278 (1.842–2.817)	<0.001	1.488 (1.187–1.865)	0.001	4.614 (2.978–7.149)	<0.001	2.907 (1.848–4.572)	<0.001

Abbreviations: TTFT, time-to-first-treatment; CSS, cancer-specific survival; HR, hazard ratio; 95% CI, 95% confidence interval; ECOG, eastern cooperative oncology group; PS, performance status; ALC, absolute lymphocytic count; Hb, haemoglobin; PLT, platelet; LDH, lactate dehydrogenase; β_2_-MG, β_2_-microglobulin; *IGHV*, immunoglobulin heavy chain variable region; HDL-C, high-density lipoprotein cholesterol; LDL-C, low-density lipoprotein cholesterol; ULN, upper limit of normal.

**Table 3:** Comparisons of C-indexes, 3-year and 5-year AUC between models and CLL-IPI in derivation and validation cohorts.

Models	CSS				TTFT			
	Derivation Cohort		Internal Validation Cohort		Derivation Cohort		Internal Validation Cohort	
	C-index (95% CI)	*p*-value	C-index (95% CI)	*p*-value	C-index (95% CI)	*p*-value	C-index (95% CI)	*p*-value
Model_Lipo-IPI_	0.838 (0.821–0.855)		0.839 (0.819–0.859)		0.687 (0.673–0.701)		0.688 (0.668–0.708)	
Model_CLL-IPI_	0.813 (0.795–0.831)	0.004	0.791 (0.764–0.818)	<0.001	0.677 (0.662–0.692)	0.093	0.676 (0.656–0.696)	0.186
CLL-IPI	0.810 (0.792–0.828)	0.006	0.792 (0.765–0.819)	0.002	0.665 (0.650–0.680)	0.002	0.670 (0.650–0.690)	0.075
	3-year AUC (95% CI)	*p*-value	3-year AUC (95% CI)	*p*-value	1-year AUC (95% CI)	*p*-value	1-year AUC (95% CI)	*p*-value
Model_Lipo-IPI_	0.890 (0.851–0.930)		0.878 (0.830–0.926)		0.746 (0.703–0.789)		0.746 (0.686–0.806)	
Model_CLL-IPI_	0.843 (0.796–0.890)	<0.001	0.819 (0.753–0.886)	<0.001	0.746 (0.704–0.789)	0.935	0.751 (0.691–0.810)	0.743
CLL-IPI	0.829 (0.778–0.881)	<0.001	0.809 (0.739–0.880)	<0.001	0.730 (0.686–0.773)	0.129	0.735 (0.674–0.795)	0.473
	5-year AUC (95% CI)	*p*-value	5-year AUC (95% CI)	*p*-value	3-year AUC (95% CI)	*p*-value	3-year AUC (95% CI)	*p*-value
Model_Lipo-IPI_	0.868 (0.823–0.914)		0.879 (0.833–0.925)		0.772 (0.728–0.817)		0.785 (0.723–0.847)	
Model_CLL-IPI_	0.841 (0.792–0.889)	0.020	0.816 (0.752–0.879)	0.001	0.761 (0.716–0.806)	0.275	0.785 (0.725–0.845)	0.999
CLL-IPI	0.835 (0.788–0.883)	0.014	0.819 (0.758–0.881)	0.002	0.739 (0.693–0.784)	0.003	0.770 (0.708–0.833)	0.360

Abbreviations: C-index, concordance index; AUC, area under the curve; CLL, chronic lymphocytic leukaemia; IPI, international prognostic index; CSS, cancer-specific survival; TTFT, time to first treatment; 95% CI, 95% confidence interval; Model_Lipo-IPI_, model generated by nomogram with cholesterol profile; Model_CLL-IPI_, model generated by nomogram without cholesterol profile.

## Data Availability

De-identified participant data that support the findings of this study are available upon reasonable request from the corresponding author after clearance by the ethical committee.

## Supplementary Materials

The supporting information can be downloaded at: https://www.mdpi.com/article/10.3390/ijms24087396/s1.
