# Supplementary material for: Low Serum Cholesterol Level Is a Significant Prognostic Factor That Improves CLL-IPI in Chronic Lymphocytic Leukaemia
Source: Int J Mol Sci. 2023 Apr 17;24(8):7396. doi: 10.3390/ijms24087396 (PMC10138885; doi:10.3390/ijms24087396)
Supplement: Supplementary file 1 [file ijms-24-07396-s001.zip › ijms-2247576-supplementary.pdf]

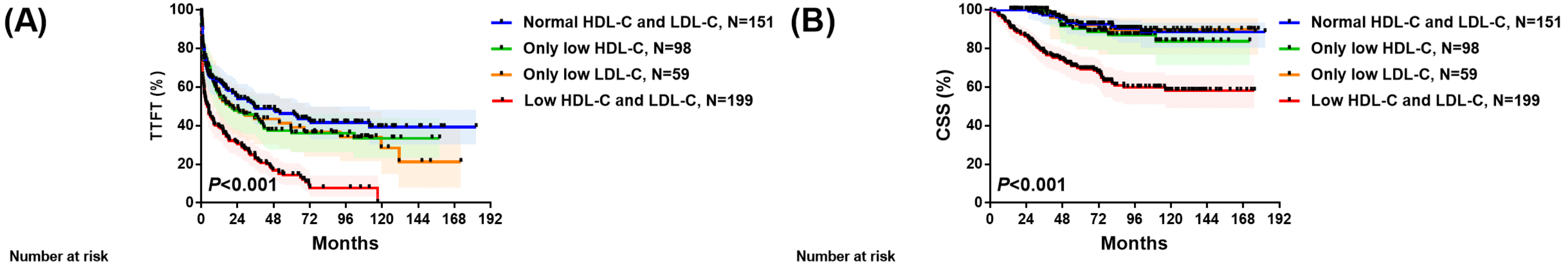

(A) Number at risk

Low HDL-C and LDL-C	151	84	75	68	67	66	66	66	66
Only low HDL-C	98	47	38	37	37	36	36	36	36
Only low LDL-C	59	29	26	23	22	21	20	20	20
Normal HDL-C and LDL-C	199	60	39	32	31	30	30	30	30

(B) Number at risk

Low HDL-C and LDL-C	151	151	145	141	139	138	138	138	138
Only low HDL-C	98	98	91	89	88	87	87	87	87
Only low LDL-C	59	59	57	55	54	54	54	54	54
Normal HDL-C and LDL-C	199	172	151	142	134	133	133	133	133

Supplemental Figure S1. Prognosis of four subgroups categorized by high-density lipoprotein cholesterol (HDL-C) and low-density lipoprotein cholesterol (LDL-C) cut-off values. **(A)** Kaplan-Meier survival curves of time-to-first-treatment (TTFT). **(B)** Kaplan-Meier survival curves of cancer-specific survival (CSS).

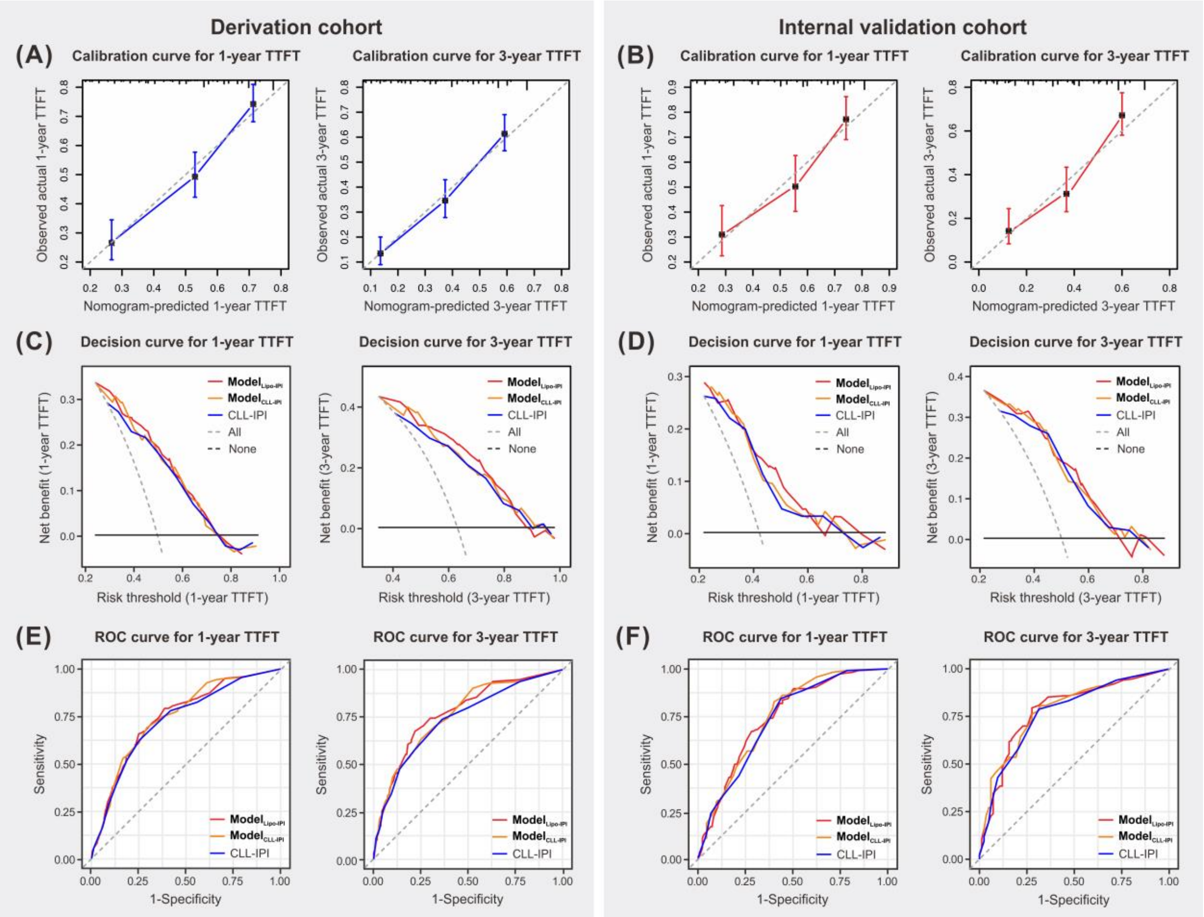

Supplemental Figure S2. Prognostic performance of Model$_{\text{Lipo-IPI}}$ for time-to-first-treatment (TTFT). **(A, B)** Calibration curves of Model$_{\text{Lipo-IPI}}$ for predicting 1-year and 3-year TTFT in the derivation **(A)** and internal validation **(B)** cohorts. **(C, D)** Decision curve analyses of different models for predicting 1-year and 3-year TTFT in the derivation **(C)** and internal validation **(D)** cohorts. **(E, F)** Receiver operating characteristic (ROC) curves of different models for predicting 1-year and 3-year TTFT in the derivation **(E)** and internal validation **(F)** cohorts. **Abbreviations:** CLL-IPI, international prognostic index for chronic lymphocytic leukaemia.

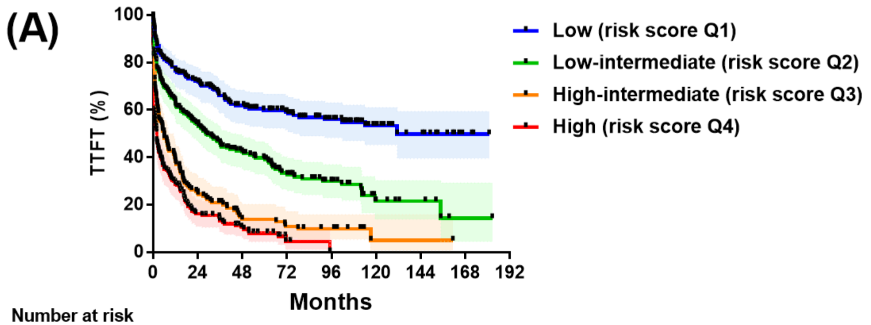

Number at risk

Risk score Q1	191	138	120	116	112	110	109	109	109
Risk score Q2	210	114	92	79	75	70	70	70	70
Risk score Q3	168	42	27	24	23	22	22	22	22
Risk score Q4	192	32	24	19	18	18	18	18	18

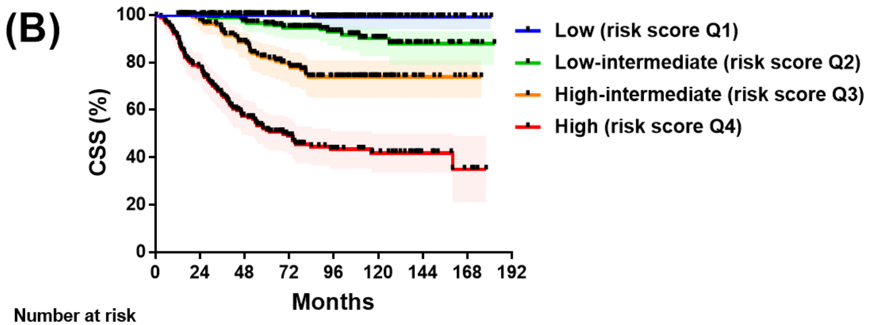

Number at risk

Risk score Q1	191	191	191	191	190	190	190	190	190
Risk score Q2	210	209	205	201	199	197	196	196	196
Risk score Q3	168	165	151	140	134	134	134	134	134
Risk score Q4	192	151	114	102	96	95	95	94	94

Supplemental Figure S3. Prognosis of four risk grades stratified by quantiles of Model$_{Lipo\text{-}IPI}$. **(A)** Kaplan-Meier survival curves of time-to-first-treatment (TTFT). **(B)** Kaplan-Meier survival curves of cancer-specific survival (CSS).

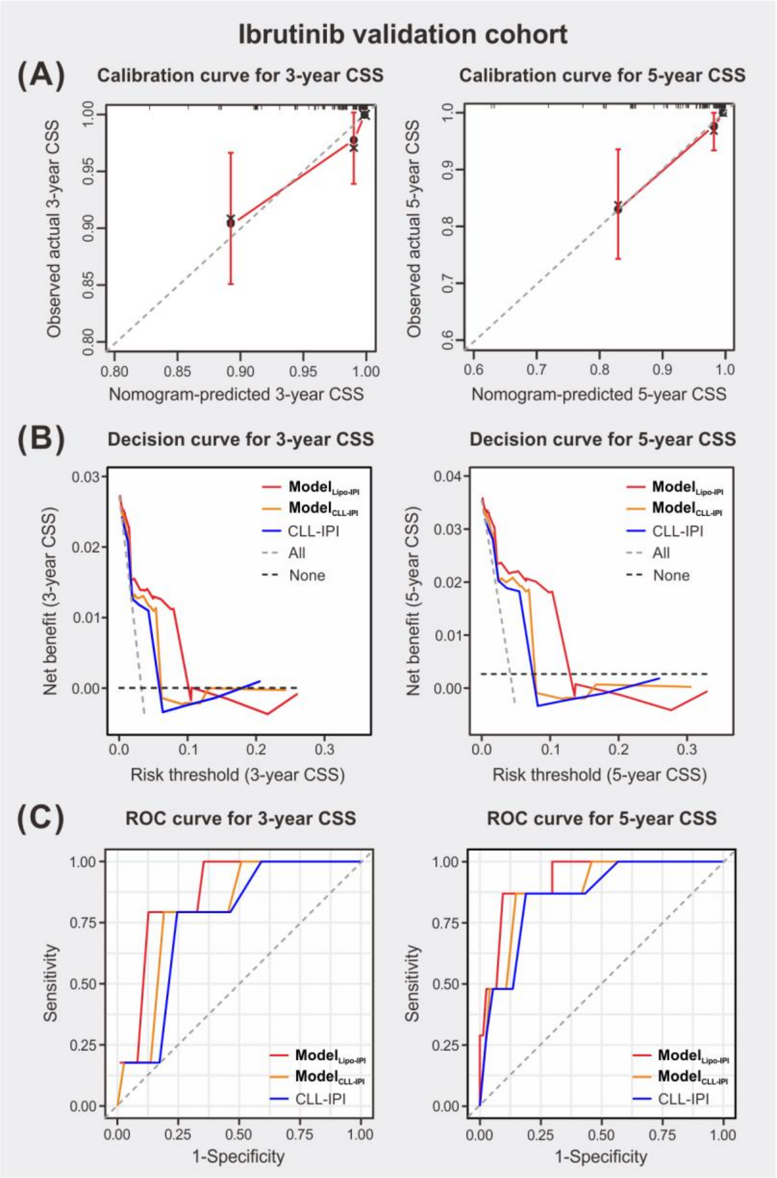

Supplemental Figure S4. Prognostic performance of Model$_{\text{Lipo-IPI}}$ for cancer-specific survival (CSS) in the ibrutinib validation cohort. **(A)** Calibration curves of Model$_{\text{Lipo-IPI}}$ for predicting 3-year and 5-year CSS. **(B)** Decision curve analyses of different models for predicting 3-year and 5-year CSS. **(C)** Receiver operating characteristic (ROC) curves of different models for predicting 3-year and 5-year CSS. **Abbreviations:** CLL-IPI, international prognostic index for chronic lymphocytic leukaemia.

Supplemental Table S1. Pairwise comparisons of four serum cholesterol groups for TTFT and CSS in the derivation cohort.

**Log-rank test (Mantel-Cox)**	**Serum cholesterol group**	**Normal HDL-C and LDL-C**		**Only low HDL-C**		**Only low LDL-C**		**Low HDL-C and LDL-C**	
		**Chi- square**	***p*-value**	**Chi- square**	***p*-value**	**Chi- square**	***p*-value**	**Chi- square**	***p*-value**
TTFT (Overall *p*<0.001)	Normal HDL-C and LDL-C	–	–	1.038	0.308	1.045	0.307	49.374	<0.001
	Only low HDL-C	1.038	0.308	–	–	0.012	0.914	25.976	<0.001
	Only low LDL-C	1.045	0.307	0.012	0.914	–	–	19.251	<0.001
	Low HDL-C and LDL-C	49.374	<0.001	25.976	<0.001	19.251	<0.001	–	–
CSS (Overall *p*<0.001)	Normal HDL-C and LDL-C	–	–	0.575	0.448	0.002	0.962	34.523	<0.001
	Only low HDL-C	0.575	0.448	–	–	0.414	0.520	18.709	<0.001
	Only low LDL-C	0.002	0.962	0.414	0.520	–	–	15.585	<0.001
	Low HDL-C and LDL-C	34.523	<0.001	18.709	<0.001	15.585	<0.001	–	–

Abbreviations: TTFT, time-to-first-treatment; CSS, cancer-specific survival; HDL-C, high-density lipoprotein cholesterol; LDL-C, low-density lipoprotein cholesterol.

Supplemental Table S2. Post-chemoimmunotherapeutic cholesterol fluctuation stratified by different treatment regimens and response to treatment.

Treatment regimes	Response to treatment	Total	Pre- or post-therapeutic	TC (mmol/L)	*p*-value	HDL-C (mmol/L)	*p*-value	LDL-C (mmol/L)	*p*-value
Fludarabine + cyclophosphamide ± rituximab	CR or PR	104	Before	3.94±1.05	0.006	0.99±0.3	<0.001	2.48±0.67	0.005
			After	4.22±0.97		1.09±0.31		2.70±0.70	
	SD or PD	79	Before	3.95±1.03	0.028	0.86±0.25	0.093	2.51±0.78	0.002
			After	3.72±1.02		0.82±0.24		2.26±0.74	
Bendamustine ± rituximab	CR or PR	28	Before	3.89±0.88	0.266	0.95±0.30	0.154	2.44±0.67	0.384
			After	4.13±1.03		1.06±0.39		2.57±0.70	
	SD or PD	9	Before	3.95±1.05	0.447	0.92±0.17	0.145	2.32±0.37	0.231
			After	3.62±0.53		0.83±0.12		2.13±0.33	
Chlorambucil ± rituximab	CR or PR	52	Before	4.09±0.90	0.453	0.93±0.26	0.114	2.47±0.66	0.128
			After	4.20±1.19		1.00±0.31		2.61±0.78	
	SD or PD	64	Before	3.99±1.03	0.005	0.94±0.28	0.001	2.51±0.71	0.027
			After	3.63±1.00		0.81±0.25		2.32±0.73	
Ibrutinib ± rituximab	CR or PR	78	Before	4.05±0.91	<0.001	0.96±0.29	<0.001	2.52±0.65	<0.001
			After	4.48±1.08		1.08±0.31		2.79±0.76	
	SD or PD	41	Before	3.90±1.04	0.065	0.93±0.20	0.708	2.53±0.84	0.036
			After	3.61±1.00		0.91±0.27		2.27±0.75	
Ibrutinib + fludarabine + cyclophosphamide + rituximab	CR or PR	18	Before	4.19±1.30	0.755	1.05±0.33	0.566	2.62±0.90	0.874
			After	4.27±1.22		1.10±0.29		2.59±0.93	
	SD or PD	5	Before	3.67±0.60	0.950	0.96±0.25	0.619	2.35±0.36	0.660
			After	3.69±1.10		0.90±0.27		2.21±0.77	
Other treatments	CR or PR	21	Before	4.08±1.12	0.013	1.03±0.34	0.104	2.50±0.88	0.071
			After	4.71±1.00		1.15±0.34		2.98±0.86	
	SD or PD	22	Before	4.00±1.11	0.034	1.00±0.31	0.004	2.46±0.89	0.028
			After	3.55±0.94		0.83±0.29		2.15±0.72	

Abbreviations: TC, total cholesterol; HDL-C, high-density lipoprotein cholesterol; LDL-C, low-density lipoprotein cholesterol; CR, complete remission; PR, partial remission; SD, stable disease; PD, progressive disease.

Supplemental Table S3. Post-chemoimmunotherapeutic cholesterol fluctuation stratified by different biological variables and response to treatment.

Biological variables	Status	Response to treatment	Total	Pre- or post-therapeutic	TC (mmol/L)	*p*-value	HDL-C (mmol/L)	*p*-value	LDL-C (mmol/L)	*p*-value
TP53 disruption	Absence	CR or PR	237	Before	4.06±1.01	<0.001	0.98±0.29	<0.001	2.54±0.67	0.001
				After	4.34±1.06		1.07±0.32		2.71±0.74	
		SD or PD	153	Before	3.99±1.04	<0.001	0.94±0.27	<0.001	2.53±0.80	<0.001
				After	3.40±1.06		0.85±0.26		2.24±0.71	
	Presence	CR or PR	64	Before	3.84±1.03	0.002	0.95±0.32	0.001	2.31±0.79	0.001
				After	4.22±1.19		1.08±0.33		2.70±0.87	
		SD or PD	67	Before	3.86±1.01	0.007	0.86±0.24	0.090	2.41±0.67	0.101
				After	3.58±1.07		0.81±0.24		2.30±0.77	
ATM deletion	Absence	CR or PR	248	Before	4.01±1.04	<0.001	0.98±0.30	<0.001	2.48±0.72	<0.001
				After	4.31±1.12		1.08±0.32		2.71±0.79	
		SD or PD	183	Before	3.94±1.03	<0.001	0.91±0.26	<0.001	2.49±0.77	<0.001
				After	3.48±1.10		0.84±0.26		2.28±0.74	
	Presence	CR or PR	53	Before	4.06±0.94	0.070	0.96±0.27	0.001	2.56±0.61	0.101
				After	4.31±0.91		1.08±0.30		2.71±0.66	
		SD or PD	37	Before	3.98±1.05	<0.001	0.92±0.28	0.013	2.53±0.76	0.002
				After	3.33±0.90		0.81±0.25		2.16±0.65	
IGHV	unmutated	CR or PR	167	Before	4.04±1.08	<0.001	0.98±0.29	<0.001	2.52±0.73	<0.001
				After	4.36±1.06		1.10±0.31		2.72±0.72	
		SD or PD	108	Before	3.92±1.07	<0.001	0.96±0.29	0.009	2.44±0.80	0.069
				After	3.47±1.08		0.89±0.25		2.31±0.71	
	mutated	CR or PR	134	Before	3.98±0.94	0.004	0.97±0.31	0.004	2.45±0.67	0.001
				After	4.25±1.12		1.05±0.33		2.69±0.82	
		SD or PD	112	Before	3.98±0.99	<0.001	0.86±0.23	<0.001	2.55±0.73	<0.001
				After	3.44±1.06		0.78±0.25		2.21±0.75	

CD38	<30%	CR or PR	224	Before	4.00±1.05	<0.001	0.97±0.31	<0.001	2.49±0.72	<0.001
				After	4.28±1.13		1.08±0.33		2.71±0.77	
		SD or PD	144	Before	3.89±0.99	<0.001	0.91±0.26	0.001	2.45±0.73	<0.001
				After	3.46±1.08		0.85±0.26		2.25±0.72	
	≥30%	CR or PR	77	Before	4.07±0.91	<0.001	0.97±0.27	<0.001	2.50±0.65	0.004
				After	4.39±0.97		1.05±0.30		2.71±0.76	
		SD or PD	76	Before	4.06±1.09	<0.001	0.91±0.27	0.003	2.59±0.83	0.001
				After	3.44±1.05		0.82±0.24		2.27±0.75	

Abbreviations: TC, total cholesterol; HDL-C, high-density lipoprotein cholesterol; LDL-C, low-density lipoprotein cholesterol; CR, complete remission; PR, partial remission; SD, stable disease; PD, progressive disease; *IGHV*, immunoglobulin heavy chain variable region.

Supplemental Table S4. Pairwise comparisons of four nomogram risk score groups for TTFT and CSS in the entire cohort.

Log-rank test (Mantel-Cox)	Risk group	Low (risk score Q1)		Low-intermediate (risk score Q2)		High-intermediate (risk score Q3)		High (risk score Q4)	
		**Chi- square**	***p*-value**	**Chi- square**	***p*-value**	**Chi- square**	***p*-value**	**Chi- square**	***p*-value**
TTFT (Overall *p*<0.001)	Low (risk score Q1)	–	–	27.125	<0.001	107.082	<0.001	163.966	<0.001
	Low-intermediate (risk score Q2)	27.125	<0.001	–	–	37.906	<0.001	86.515	<0.001
	High-intermediate (risk score Q3)	107.082	<0.001	37.906	<0.001	–	–	8.306	0.004
	High (risk score Q4)	163.966	<0.001	86.515	<0.001	8.306	0.004	–	–
CSS (Overall *p*<0.001)	Low (risk score Q1)	–	–	11.312	0.001	43.024	<0.001	134.991	<0.001
	Low-intermediate (risk score Q2)	11.312	0.001	–	–	17.145	<0.001	108.364	<0.001
	High-intermediate (risk score Q3)	43.024	<0.001	17.145	<0.001	–	–	41.233	<0.001
	High (risk score Q4)	134.991	<0.001	108.364	<0.001	41.233	<0.001	–	–

Abbreviations: TTFT, time-to-first-treatment; CSS, cancer-specific survival.

Supplemental Table S5. Univariate and multivariate analyses of CSS including regimen types in treated patients of the entire cohort.

Variables	CSS			
	Univariate analyses		Multivariate analyses	
	HR (95% CI)	*p*-value	HR (95% CI)	*p*-value
Male	1.165 (0.796−1.706)	0.432	−	−
Age >65 years	1.604 (1.124−2.288)	0.009	1.577 (1.103−2.255)	0.013
Binet B/C	3.472 (1.762−6.842)	<0.001	1.423 (0.713−2.841)	0.317
ECOG PS >1	1.435 (0.897−2.295)	0.132		
B symptoms	0.881 (0.582−1.335)	0.550	−	−
Novel targeted therapies*	0.100 (0.037−0.272)	<0.001	0.095 (0.035−0.257)	<0.001
ALC >50×10^9/L	1.184 (0.806−1.738)	0.390	−	−
Hb <100 g/L	1.745 (1.211−2.515)	0.003	0.999 (0.661−1.511)	0.998
PLT <100×10^9/L	1.809 (1.274−2.570)	0.001	1.150 (0.793−1.668)	0.462
LDH >ULN (271 U/L)	1.750 (1.214−2.522)	0.003	1.022 (0.679−1.539)	0.916
β_2-MG >3.50 mg/L	2.073 (1.427−3.011)	<0.001	1.209 (0.823−1.775)	0.334
TP53 disruption	4.416 (3.102−6.287)	<0.001	3.221 (2.255−4.600)	<0.001
ATM deletion	0.707 (0.418−1.196)	0.196	−	−
IGHV unmutated	3.053 (2.082−4.477)	<0.001	3.239 (2.199−4.772)	<0.001
CD38 ≥30%	1.453 (1.006−2.099)	0.047	1.103 (0.755−1.614)	0.612
Low HDL-C and LDL-C	3.735 (2.517−5.543)	<0.001	3.437 (2.302−5.131)	<0.001

Abbreviations: CSS, cancer-specific survival; HR, hazard ratio; 95% CI, 95% confidence interval; ECOG, eastern cooperative oncology group; PS, performance status; ALC, absolute lymphocytic count; Hb, haemoglobin; PLT, platelet; LDH, lactate dehydrogenase; β_2-MG, β_2-microglobulin; *IGHV*, immunoglobulin heavy chain variable region; HDL-C, high-density lipoprotein cholesterol; LDL-C, low-density lipoprotein cholesterol; ULN, upper limit of normal.

*Novel targeted therapies refer to ibrutinib ± rituximab, ibrutinib + fludarabine + cyclophosphamide + rituximab and part of the other treatments.

Supplemental Table S6. Comparisons of C-indexes, 3-year and 5-year AUC between models and CLL-IPI in the **ibrutinib** validation cohort.

**Models**	**CSS**	
	**Ibrutinib validation cohort**	
	**C-index (95% CI)**	***p*-value**
$Model_{Lipo\text{-}IPI}$	0.889 (0.846−0.932)	
$Model_{CLL\text{-}IPI}$	0.835 (0.776−0.894)	**0.002**
CLL-IPI	0.807 (0.742−0.872)	**0.001**
	**3-year AUC (95% CI)**	***p*-value**
$Model_{Lipo\text{-}IPI}$	0.864 (0.757−0.972)	
$Model_{CLL\text{-}IPI}$	0.797 (0.653−0.942)	**0.005**
CLL-IPI	0.760 (0.606−0.914)	**<0.001**
	**5-year AUC (95% CI)**	***p*-value**
$Model_{Lipo\text{-}IPI}$	0.926 (0.852−1.000)	
$Model_{CLL\text{-}IPI}$	0.882 (0.774−0.990)	**0.028**
CLL-IPI	0.860 (0.737−0.982)	**0.015**

Abbreviations: C-index, concordance index; AUC, area under the curve; CLL, chronic lymphocytic leukaemia; IPI, international prognostic index; CSS, cancer-specific survival; 95% CI, 95% confidence interval; $Model_{Lipo\text{-}IPI}$, model generated by nomogram with cholesterol profile; $Model_{CLL\text{-}IPI}$, model generated by nomogram without cholesterol profile.
